# Nuclear translocation of fibroblast growth factor-2 (FGF2) is regulated by Karyopherin-β2 and Ran GTPase in human glioblastoma cells

**DOI:** 10.18632/oncotarget.4097

**Published:** 2015-05-27

**Authors:** Feng Wang, Lijun Yang, Lin Shi, Qian Li, Gengshen Zhang, Jianliang Wu, Jun Zheng, Baohua Jiao

**Affiliations:** ^1^ Department of Neurosurgery, The Second Hospital of Hebei Medical University, Shijiazhuang 050000, China; ^2^ Department of Neurosurgery, The Second Hospital of Baoding City, Baoding 071051, China; ^3^ Department of Physiology, Hebei Medical University, Shijiazhuang 050000, China

**Keywords:** fibroblast growth factor (FGF), glioblastoma, nuclear translocation, phosphatase and tensin homolog (PTEN), small GTPase

## Abstract

Human glioblastoma multiforme (GBM) is the most malignant tumor of the central nervous system (CNS). Fibroblast growth factor-2 (FGF2) belongs to the FGF superfamily and functions as a potential oncoprotein in GBM. FGF2 has low molecular weight (18K) and high molecular weight (HMW) isoforms. Nuclear accumulation of HMW-FGF2 strongly promotes glioblastoma cell proliferation, yet mechanism governing such cellular distribution remains unexplored. We investigated the mechanisms regulating FGF2 cellular localization in T98G human brain glioblastoma cells. We found HMW-FGF2, but not 18K-FGF2, is primarily located in the nucleus and interacts with nuclear transport protein Karyopherin-β2/Transportin (Kapβ2). SiRNA-directed Kapβ2 knockdown significantly reduced HMW-FGF2′s nuclear translocation. Moreover, inhibiting Ran GTPase activity also resulted in decreased HMW-FGF2 nuclear accumulation. Proliferation of T98G cells is greatly enhanced with transfections HMW-FGF2. Decreased PTEN expression and activated Akt signaling were observed upon HMW-FGF2 overexpression and might mediate pro-survival effect of FGF2. Interestingly, addition of nuclear localization signal (NLS) to 18K-FGF2 forced its nuclear import and dramatically increased cell proliferation and Akt activation. These findings demonstrated for the first time the molecular mechanisms for FGF2′s nuclear import, which promotes GBM cell proliferation and survival, providing novel insights to the development of GBM treatments.

## INTRODUCTION

Fibroblast growth factors (FGFs) superfamily consists of 22 FGF genes in mice and humans [1]. FGFs drive crucial biological functions such as early embryonic development and organogenesis via binding to FGF receptors (FGFRs) [2, 3]. FGF2, also known as basic FGF, belongs to FGF superfamily and is recognized as an pro-angiogenic factor for new blood vessel formation and wound repair [4]. Meanwhile, emerging evidence suggests that FGF2 functions as a potential oncogenic protein driving a variety of tumor malignancies. Human melanoma commonly expresses high levels of FGFR1 and FGF2. Antisense-mediated inhibition of FGF2 or FGFR1 led to growth regression of xenografts formed by human melanoma cells [5]. Up-regulated FGF2-FGFR1 signaling is also implicated in the pathogenesis of prostate cancer, small cell lung cancer and glioblastoma multiforme (GBM) [6, 7]. Previous study demonstrated that high FGF2 mRNA expression is observed in over 94% of human glioblastomas [8]. It's been shown that high FGF2 expression promotes human glioma's malignancy [9]. Studies suggested that FGF2 also exerts anti-apoptotic function through up-regulating anti-apoptotic genes BCL-2 and BCL-X_L_, making tumor cells resistant to chemotherapy [10, 11].

In human, FGF2 has five isoforms (18, 22, 22.5, 24 and 34 kDa), which are produced by alternative initiation of translation on the FGF2 mRNA [12]. The translation of low molecular weight 18 kDa FGF2 is initiated from the classic Kozak AUG start codon. This 18K-FGF2 is made up of 155 amino acids, which is also the core domain for all FGF2 isoforms [13]. The other four FGF2 isoforms are generally recognized as HMW FGF2s and are translated from alternative upstream CUG start codons. Previous studies revealed that HMW-FGF2 is primarily localized in nucleus whereas 18K-FGF2 resides in cytosol, and the nuclear FGF2 accumulation is associated with proliferation of human glioma cells [14, 15]. Mechanisms regulating such divergent cellular distribution of different FGF2 isoforms remain unclear.

In this study, we investigated how HMW-FGF2 is translocated into the nucleus, as well as the impact of nuclear FGF2 on proliferation and survival of the T98G GBM cell line. Transfected HMW-FGF2 is found to associate with Karyopherin-β2 (Kapβ2), that belongs to the nuclear transport karyopherin protein family and also known as Transportin, functioning to transport macromolecule cargos between cytoplasm and nucleus [16]. A small GTPase Ran, which regulates the Kapβ2-cargo interaction, is also involved in mediating the nuclear localization of HMW-FGF2. Compared to 18K-FGF2, HMW-FGF2 overexpression in T98G cells resulted in a significantly higher proliferation rate. Together, our study demonstrated for the first time that Kapβ2 and Ran GTPase synergistically regulate the nuclear import of HMW-FGF2, which plays an important role in GBM cell proliferation.

## RESULTS

**Figure 1:**
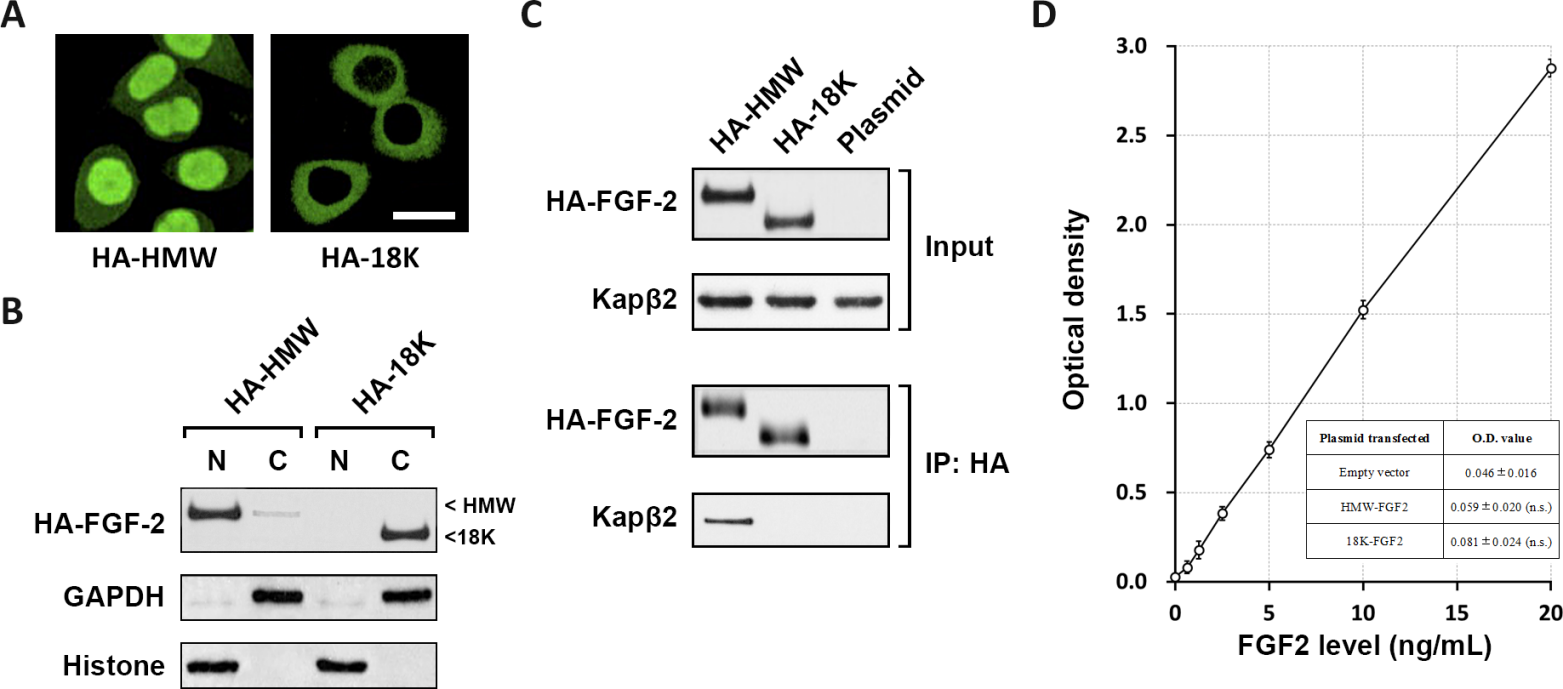
HMW FGF2 localizes in the nucleus and associates with Kapβ2 **A.** T98G cells transfected with the two HA-FGF2 isoforms (HA-HMW and HA-18K) were immunostained by HA antibody. Scale bar 10 μm. **B.** Nuclear and cytosolic fractionation of T98G cells transfected with HA-FGF2 isoforms (HA-HMW and HA-18K) were subjected to western blot analysis using HA antibody. GAPDH and Histone H3 were used as marker for cytosolic **C.** and nuclear (N) fractions respectively. C. Whole cell lysates were extracted from T98G cells transfected with either HA-HMW or HA-18K, as well as plasmid control, and subjected to immunoprecipitation with HA antibody, followed by western blot analysis using antibodies against HA and Kapβ2. Top panels: input; bottom panels: IP. **D.** Levels of FGF2 in the media of indicated experimental groups were measured using ELISA by plotting to curve of known FGF2 concentrations. Data are shown as mean ± SEM of three independent experiments. n.s. not significant vs empty vector.

Five known FGF2 isoforms can be classified into low molecular weight FGF2 (18K) and high molecular weight (HMW) FGF2, which are reported to localize either in the nucleus or cytosol [17]. To study the localization of both 18K and HMW FGF2 in glioblastoma cells, T98G cell line derived from human glioblastoma was transfected with HA-tagged constructs expressing HMW-FGF2 (HA-HMW) or 18K-FGF2 (HA-18K). Immunostaining against HA showed that HA-HMW is primarily located in the nucleus while HA-18K mainly resides in the cytosol (Fig. 1A). Even though there are three HMW FGF2 isoforms that display distinct molecular weights (22, 24, 34 kDa), the transfection of HMW-FGF2 construct led to the expression of a pure HMW-FGF2 population because of that HA tag addition at the N-terminal eliminates the alternative CTG start codon for HMW-FGF2 translation. This specific expression pattern of HMW-FGF2 excludes experimental variance and provides a favorable assay condition. Western blot analysis using nuclear and cytosolic fractionations extracted from HWM or 18K FGF2-transfected T98G cells revealed that almost all of overexpressed 18K-FGF2 is located in the cytosol. The majority of HMW-FGF2 is nucleus-localized, with a small amount of cytosolic HA-HMW FGF2 (Fig. 1B).

The transport of proteins between nucleus and cytoplasm is mediated by nuclear transport factors such as Karyopherin-β (Kapβ) family proteins that recognize nuclear localization signals (NLS) [18]. To confirm whether Kapβ2 is involved in mediating the preferential nuclear localization of HA-HMW FGF2, which potentially contains a non-classical arginine-glycine-rich NLS (see discussion for details), T98G cells were transfected with HA-HMW, HA-18K and empty plasmid before immunoprecipitation (IP) assay was performed to examine the association between Kapβ2 and transfected FGF2. Analysis using input cell lysate showed desired FGF2 expressions with corresponding FGF2 constructs. IP results indicated that HMW-FGF2, not the 18K-FGF2, physically interacted with Kapβ2 (Fig. 1C).

It is reported that HMW-FGF2 is not secreted and regulates multiple cellular events in an intracrine manner [19]. To determine whether the overexpressed FGF2 in our system functions in the same fashion, we measured the FGF2 concentrations in the medium collected from FGF-2-overexpressed T98G cells. First, using a series of media with known FGF2 concentrations as reference, a series of OD reading were obtained by ELISA (Supplementary Table 1), which were then plotted to the known FGF2 concentrations, to draw a linear curve (Fig. 1D). Next, also by ELISA, we measured the OD readings from culture media of cells transfected with empty vector, HMW-FGF2 and 18K-FGF2 (Supplementary Table 2). Finally with the linear curve, we could calculate the FGF2 concentrations in the culture media of cells transfected with empty vector, HMW-FGF2 and 18K-FGF2 from their respective OD readings. ELISA analysis showed that the FGF2 concentrations in HMW-FGF2 or 18K-FGF2 transfected cell medium are below 0.5 ng/ml and are not significantly different from the one in empty vector-transfected cells, indicating that the overexpressed FGF2 is not secreted (Fig. 1D and Supplementary tables 1 and 2).

**Figure 2:**
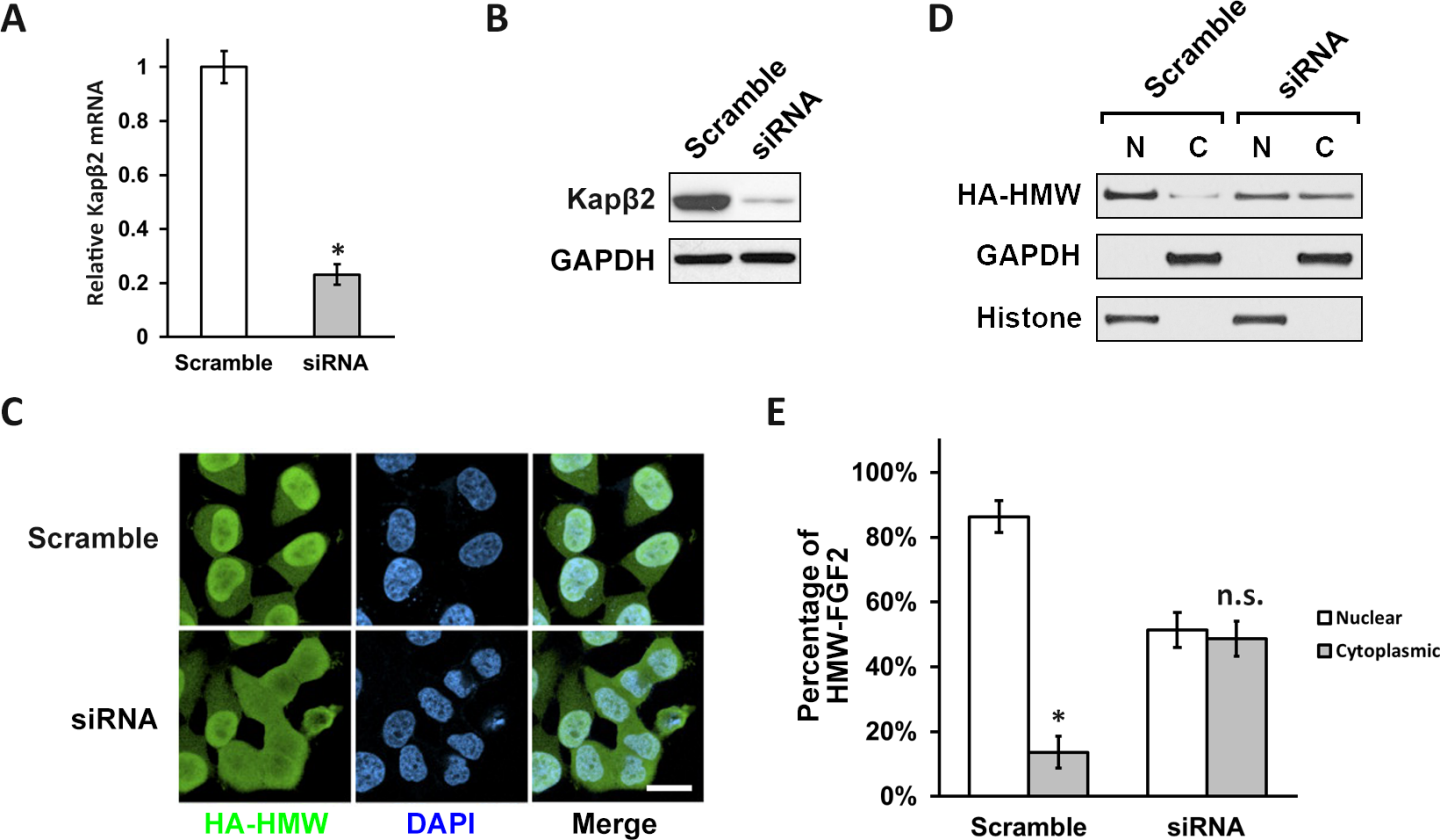
Kapβ2 is required for efficient nuclear translocation of HMW FGF2 **A and B.** T98G cells were transfected with scramble siRNA and siRNA against Kapβ2 mRNA. Kapβ2 mRNA and protein levels were examined with RT-PCR (A) and western blot (B) respectively. Data are shown as mean ± SEM of three independent experiments; *P < 0.05 versus control. **C.** T98G cells expressing HMW FGF2 (HA-HMW) were transfected with scramble siRNA or siRNA against Kapβ2, and subjected to immunostaining by HA antibody (green). Nuclei were stained by DAPI (blue). Scale bar 10 μm. **D.** T98G cells expressing HMW FGF2 were transfected with scramble siRNA or siRNA against Kapβ2, and subjected to fractionation. Nuclear (N) and cytosolic (C) fractions were analyzed by western blot using antibodies against HA to examine the subcellular distribution of HMW FGF2 protein. GAPDH and Histone H3 were used as marker for cytosolic (C) and nuclear (N) fractions respectively. **E.** Quantifications of the fluorescence signal of HMW-FGF2 in panel (C) Data are shown as mean ± SEM of three independent experiments; *P < 0.05 versus nuclear; n.s. not significant vs nuclear.

Next, we sought to investigate whether Kapβ2 is required for HMW-FGF2′s nuclear localization. Two independent siRNA sequences were used to knockdown Kapβ2 in T98G cells. Both siRNA1 and siRNA2 against Kapβ2 achieved approximately 80% knockdown in mRNA and protein levels while siRNA1 showed slightly higher but not significant efficiency (Fig. 2A and 2B). T98G cells were then co-transfected with siRNA1 and HA-HMW FGF2 followed by immunostaining using antibody against HA. Staining analysis showed that Kapβ2 knockdown blocked the nuclear transport and led to a uniform subcellular distribution of HMW-FGF2 (Fig. 2C). Quantification of fluorescent signal intensity showed that the percentage ration of nuclear to cytoplasmic HMW-FGF2 is 85% vs. 15% in Scramble group, whereas in siRNA group the difference of the ratio is not significant (approximately 51% vs. 49%) (Fig. 2E). Nuclear and cytosolic fractionations were isolated from siRNA1 and HA-HMW FGF2 co-transfected T98G cells and subjected to western blotting analysis for HMW-FGF2. Similar to immunostaining data, the immunoblot results showed that the nuclear HMW-FGF2 level was significantly reduced upon Kapβ2 knockdown (Fig. 2D). The same experiments were also performed using siRNA2, and the results were almost identical (data not shown). Together, these data indicate that Kapβ2 is required to mediate the nuclear localization of HMW-FGF2.

**Figure 3:**
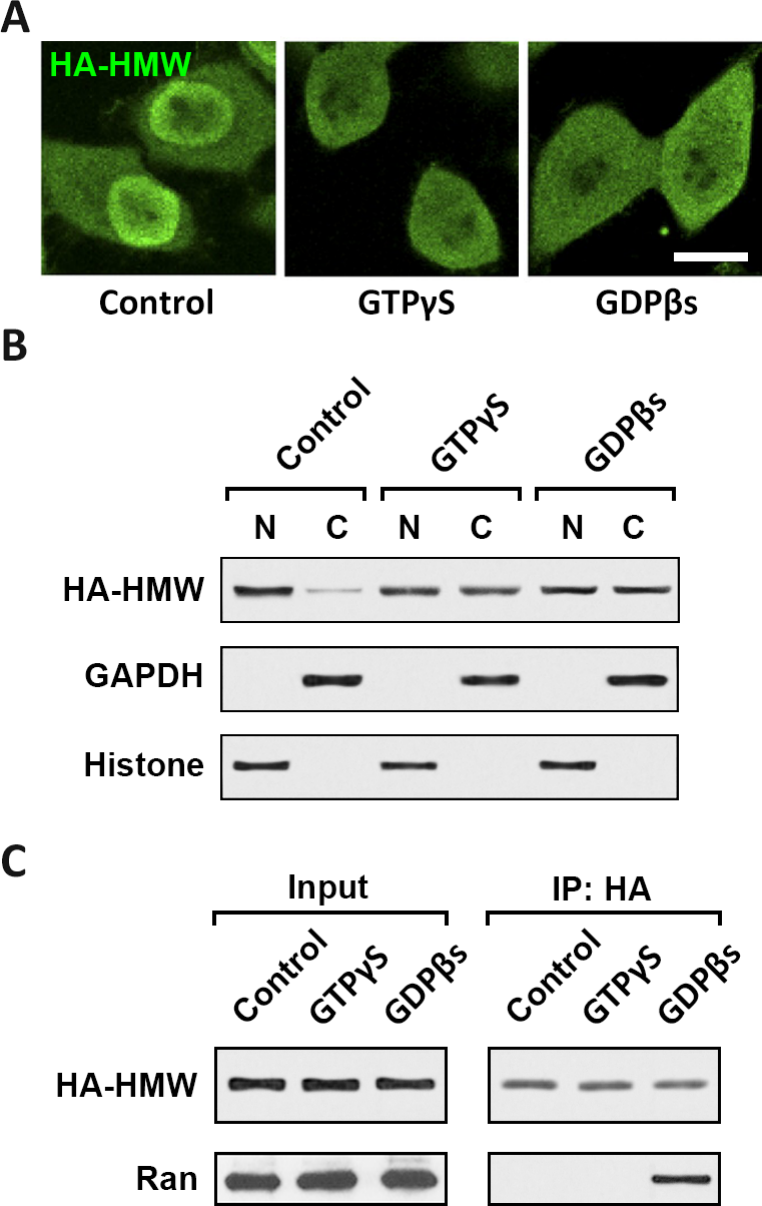
Nuclear translocation of HMW FGF2 requires Ran GTPase activity **A.** T98G cells expressing HMW FGF2 were treated with 0.1 nM either GTPγS or GDPβs, as well as control, and subjected to immunostaining by HA antibody (green). Nuclei were stained by DAPI (blue). Scale bar 10 μm. **B.** T98G cells as treated in A were also subjected to fractionation. Nuclear (N) and cytosolic **C.** fractions were analyzed by western blot using antibodies against HA to examine the subcellular distribution of HMW FGF2 protein. GAPDH and Histone H3 were used as marker for cytosolic (C) and nuclear (N) fractions respectively. C. Whole cell lysates were extracted from T98G cells as treated in A, and subjected to immunoprecipitation with HA antibody, followed by western blot analysis using antibodies against HA and Ran.

Kapβs bind to proteins in the nuclear pore complex (NPC) and facilitate the nuclear transport of Kapβ-cargo complex [20]. The Kapβ2 and cargo interaction, as well as nuclear-cytoplasmic transportation are regulated by Ran GTPase nucleotide cycle [18]. To confirm the involvement of Ran GTPase in the Kapβ2-HMW-FGF2 nuclear transport, we employed treatments of GTPγS and GDPβS. GTPγS is a GTP analogue that is non-hydrolyzable and keeps Ran in GTP-bound state, whereas GDPβS is a GDP analogue that cannot be phosphorylated and locks Ran in inactive GDP-bound state. Both treatments block GTPase activity and the GTP-GDP cycle [21]. Immunostaining against HA showed that both GTPγS and GDPβS treatments reduced HMW-FGF2 nuclear accumulation in T98G cells overexpressing HMW-FGF2 (Fig. 3A). Western blot analysis using nuclear and cytoplasmic fractions from HWM-FGF2-expressing T98G cells further confirmed that inhibiting Ran GTPase activity blocks HMW-FGF2 nuclear transport (Fig. 3B). Immunoprecipitation results showed that Ran interacts with HMW-FGF2 upon treatment of GDPβS, but not GTPγS, suggesting Ran GDP, instead of Ran GTP, binds to FGF2 in the cytoplasm and facilitates the transport into nucleus, where Ran GTP is concentrated in the nucleus and mediates the FGF2 release in the nucleoplasm (Fig. 3C).

**Figure 4:**
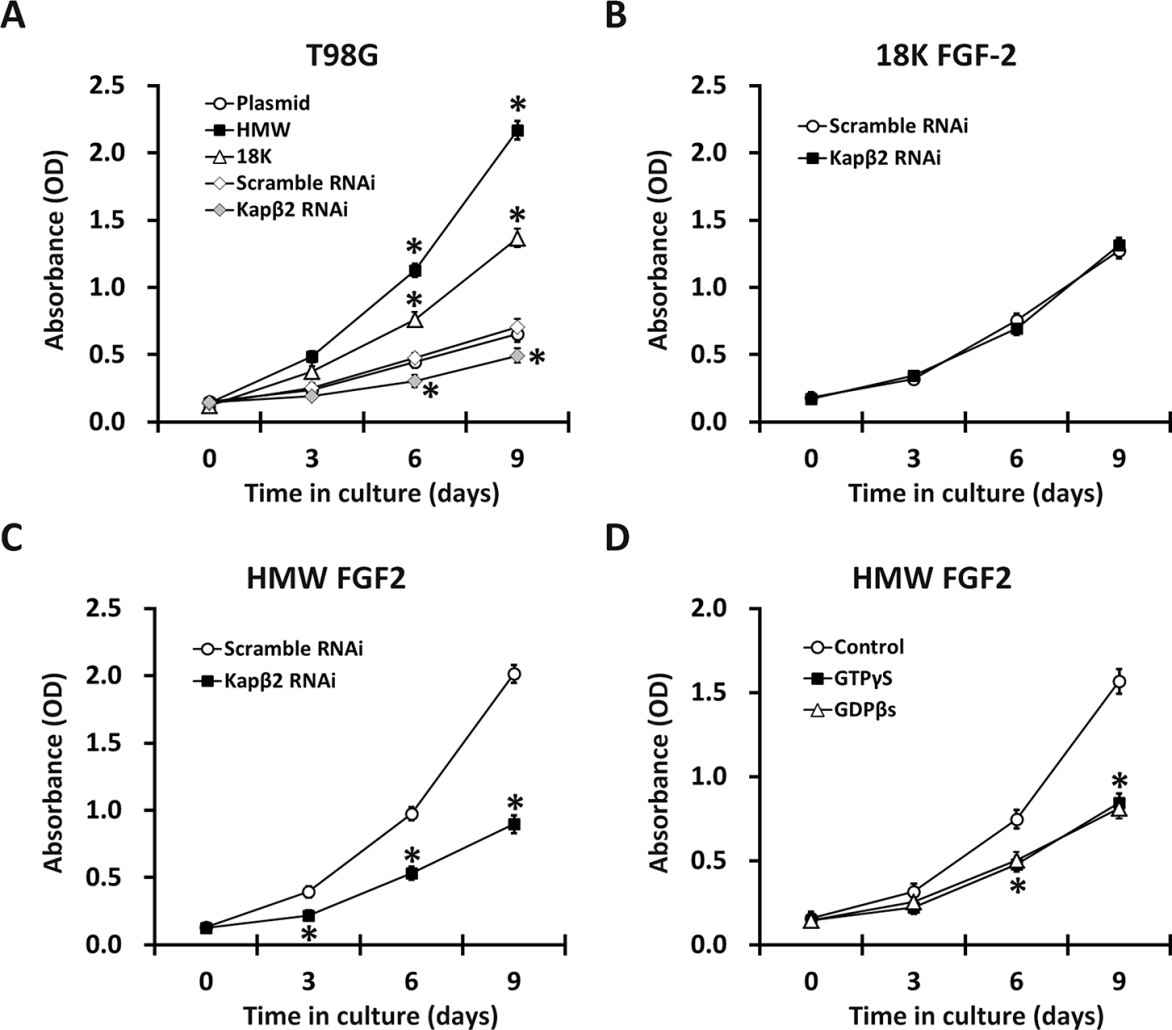
Proliferation of T98G cells expressing either HMW or 18K FGF2 subjected to RNAi against Kapβ2 (B and C) or GTPγS/GDPβs treatment (D) were measured by MTT assays **A.** Growth of T98G cells expressing HMW FGF2, 18K FGF2 or empty plasmid, as well as subjected to RNAi against Kapβ2 vs scramble RNAi. **B.** Growth of T98G cells expressing 18K FGF2 were subjected to RNAi against Kapβ2 vs scramble RNAi. **C.** Growth of T98G cells expressing HMW FGF2 were subjected to RNAi against Kapβ2 vs scramble RNAi. **D.** Growth of T98G cells expressing HMW FGF2 were treated with GTPγS or GDPβs as well as control treatment. Data are shown as mean ± SEM of three independent experiments; *P < 0.05 versus respective control.

**Figure 5:**
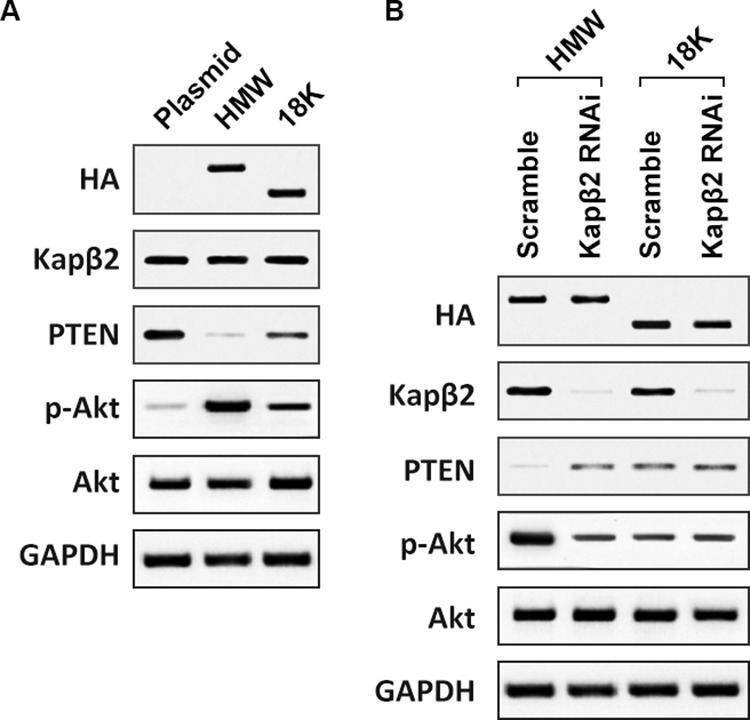
Nuclear HMW FGF2 activates Akt signaling pathway **A.** T98G cells were transfected with empty plasmid, HMW FGF2 or 18K-FGF2 respectively. Whole cell lysates were collected and subjected to western blot analysis using antibodies against HA, Kapβ2, PTEN, Akt and phosphorylated Akt (p-Akt). **B.** T98G cells expressing HMW FGF2 were transfected with scramble siRNA or siRNA against Kapβ2. Whole cell lysates were collected and subjected to western blot analysis using antibodies against HA, Kapβ2, PTEN, Akt and phosphorylated Akt (p-Akt). GAPDH was used as loading control. GAPDH was used as loading control.

FGF2 signaling drives a wide range of oncogenic events such as proliferation, migration and survival in multiple cancer cell types [3, 22]. We next evaluated the role of FGF2 in the tumorigenic potential of T98G cells. We first showed that overexpression of both HMW-FGF2 and 18K-FGF2 led to increased cell proliferation while the effect of HMW-FGF2 is more profound. Kapβ2 knockdown in native T98G cells led to reduced proliferation, indicating that Kapβ2 is required for maintenance of cell growth at the basal state (Fig. 4A). Next, we knocked down expression of Kapβ2 in HMW or 18K FGF2-expressing T98G cells using siRNA and found that Kapβ2 inhibition resulted in dramatically decreased proliferation in HMW FGF2-expressing cells. However, the proliferation of T98G cells expressing 18K-FGF2 was not affected by Kapβ2 knockdown (Fig. 4B and 4C). Similar with siRNA-mediated Kapβ2 knockdown, inhibiting Ran GTPase activity and FGF2 nuclear transport by GTPγS or GDPβS treatments significantly reduced proliferation rate in HMW-FGF2-overexpressing T98G cells (Fig. 4D), indicating that nucleus-localized HMW-FGF2 plays a crucial role in facilitating cell proliferation. The above effects on T98G cell proliferation were not due to changes in FGF2 expressions under those culturing conditions, since the protein levels of both FGF2 isoforms were found to be almost constant in the same experimental conditions (Fig. 3C and Fig. 5).

To further explore molecular mechanisms responsible for enhanced proliferation by FGF2, we focused on the PI3K/AKT signaling cascade, which is the major mitogenic pathway in response to growth factor signaling [23]. Of note, although PTEN in T98G cells is mutated [24], the L42R mutation does not affect the lipid phosphatase activity of PTEN [25], it is still functional in its ability to suppress activation of Akt [26], as evident by reported negative correlations between PTEN and p-Akt levels in T98G cells [27]. Our results showed that overexpression of 18K-FGF2 resulted in reduced PTEN expression and HMW-FGF2 overexpression inhibit PTEN level to a further extent (Fig. 5A). As a result, p-Akt level was markedly increased with FGF2 overexpression, indicating that PTEN/p-Akt cascade might mediate the enhanced cell proliferation, downstream of FGF2 signaling. Consistent with previous finding that HMW-FGF2 overexpression imposes enhanced effect on proliferation, the Akt activation was more pronounced with HMW-FGF2 overexpression than that with 18K-FGF2 transfection. Kapβ2 knockdown in HMW-FGF2-expressing T98G cells gave rise to elevated PTEN and declined p-AKT levels, suggesting the Kapβ2-mediated HMW-FGF2 nuclear translocation is indispensable for the downstream Akt activation. On the contrary, Kapβ2 knockdown in 18K-FGF2-expressing T98G cells had no effect on the levels of either PTEN or p-AKT (Fig. 5B). Moreover, GTPγS/GDPβs treatments were able to suppress Akt activation in T98G cells transfected with HMW-FGF2 (Data not shown).

**Figure 6:**
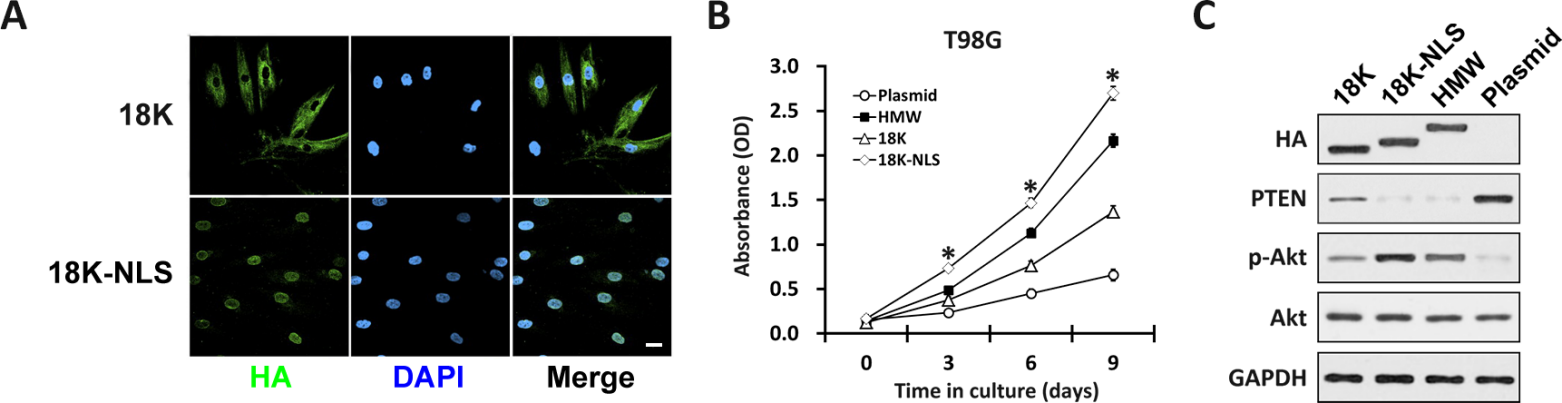
Forcing 18K FGF2 into the nucleus with classical NLS **A.** T98G cells transfected with either 18K or 18K-NLS FGF2 were immunostained by HA antibody (green). Nuclei were stained by DAPI (blue). Scale bar 10 μm. **B.** T98G cells transfected with HMW, 18K, 18K-NLS FGF2 or empty plasmid were subjected to MTT assay. Data are shown as mean ± SEM of three independent experiments; *P < 0.05 18K-NLS versus 18K and HMW. **C.** Whole cell lysates were collected from T98G cells as in B, followed by western blot analysis using antibodies against HA, PTEN, Akt and phosphorylated Akt (p-Akt). GAPDH was used as loading control. Notice the slight shift in mobility in HA-18K-FGF2 caused by the NLS.

We have demonstrated that nucleus-localized HMW-FGF2, but not cytoplasmic 18K-FGF2, is capable of activating the mitogenic AKT signaling and facilitating cell proliferation in T98G cells. Next, we hypothesize that the additional peptide sequence on HMW-FGF2 functions as a classic nuclear localization signal (NLS) to assist the nuclear transport of HMW-FGF2, which further activate its downstream molecular events. To test this hypothesis, we genetically engineered the construct expressing 18K-FGF2 that is tagged by the classic lysine-rich NLS, PKKKRKV, and transfected it into T98G cells [28]. Plasmid 18K-FGF2 served as control. Interestingly, we discovered that additional NLS sequence forced 18K-NLS-FGF2 translocate into the nucleus with undetectable cytosolic level, whereas 18K-FGF2 is dominantly cytoplasm-localized (Fig. 6A). HMW, 18K, 18K-NLS-FGF2 and empty plasmid control were transfected into T98G cells. Subsequent growth curve analysis revealed that the proliferation rate of nuclear 18K-NLS-FGF2-transfected T98G cells is significantly higher than that of other FGF2 forms-overexpressing cells, specifically HMW-FGF2-transfected T98G cells (Fig. 6B). This might due to that a small portion of HMW-FGF2 still resides within the cytoplasm, compromising the full utilization of overexpressed HMW-FGF2 (Fig. 1B). Western blot analysis showed 18K-NLS-FGF2 and HMW-FGF2 transfection led to significantly decreased PTEN expression, accompanied by increased p-Akt level. 18K-FGF2 overexpression also reduced PTEN expression, but to a much lesser extent (Fig. 6C). Taken together, the above analyses confirmed our hypothesis that the impact of HMW-FGF2 on GBM's cell proliferation is achieved via FGF2′s nuclear translocation, which is cooperatively regulated by NLS, Kapβ2 and Ran GTPase.

## DISCUSSION

In the present study, we investigated the role of FGF2, in the regulation of proliferation and survival of GBM tumor cells. Broadly, FGF2 is found to enhance the GBM cell line, T98G's proliferation and survival. In our established working model, the high molecular isoform, HMW-FGF2 exerts such effect via translocating into nucleus, a process that is coordinated by a combined action of Kapβ2, a member of nuclear transport factor family, and Ran GTPase, a GTP-binding nuclear protein involved in nuclear-cytoplasmic cargo transportation [29]. Subsequent activation of PTEN/PI3K/Akt signaling cascade upon nuclear entry of FGF2 is found to account for the increased cellular mitogenic activity. Kapβ2 knockdown or Ran GTPase's activity blockade were shown to abolish HMW-FGF2′s nuclear transport and suppress T98G's proliferation. However, the low molecular weight FGF2 isoform that is dominantly localized in cytosol, 18K-FGF2 exhibits less stimulatory effect towards cell proliferation compared to HMW-FGF2. Genetic and pharmacological manipulations of Kapβ2 or Ran GTPase did not affect proliferation of T98G cells with 18K-FGF2 overexpression. Finally, forced nuclear entry of 18K-FGF2 by classic NLS addition to the native 18K-FGF2 protein remarkably enhanced cell proliferation. These analyses demonstrated for the first time that Kapβ2 and Ran GTPase collectively facilitate the nuclear translocation of HMW-FGF2, which further potentiate the proliferation and survival of GBM tumor cells via activation of FGF2-downstream PTEN/Akt pathway.

FGF2 and epidermal growth factor (EGF) are not included in the cell culture medium used in this study for the purpose of eliminating the impact of these growth factors on MTT assay using T98G cells with FGF2 overexpression. Under our established *in vitro* culture condition, GBM tumor cells can still undergo self-renewal and form neurospheres even though proliferation is slightly compromised, which is further confirmed by previous reports [30]. Therefore, the experimental condition perfectly serves the purpose of this study and provides unbiased evidence.

Fibroblast growth factors (FGFs) transmit signal through FGF receptors and control a wide range of biological activities ranging from cell proliferation, survival to migration and differentiation [31–34]. FGF signaling is also tightly associated with fundamental developmental events, including mesoderm pattern formation in early embryo and multiple organ development [2, 3]. There is a large body of evidence suggesting aberrant FGF signaling in the pathogenesis of multiple cancer types, of which glioblastoma multiforme (GBM) is the most malignant tumor in central nervous system, with a median survival rate of less than two years and a lack of effective treatments [35]. Dysregulated FGF signaling drives tumor growth via promoting cancer cell survival and proliferation [36]. Interestingly, GBM is associated with nuclear accumulation of HMW-FGF2 [15]. The FGF superfamily consists of 22 different FGF genes, several of which have been shown to localize in the nucleus [1]. FGF8 could be translocated to the nucleus and function as a transcription factor in NIH3T3 cells, neural tube cells, as well as in mouse embryonic neural tube [37]. Moreover, it has been reported that HMW-FGF2 isoforms are not secreted from the cells. Instead, they function in an intracrine manner to mediate various cellular activities such as survival, proliferation and invasion [38, 39]. In agreement with the established results, we found that the overexpressed FGF2 are not secreted in our system and functions in a similar fashion. Our data also indicated that the additional peptide sequence on HMW-FGF2 functions as a NLS and plays a crucial role in facilitating the nuclear entry of HMW-FGF2. Interestingly, a previous study on FGF10 demonstrated that a NLS sequence on FGF10 is responsible for its dominant nuclear localization. However, genetically engineered FGF10 lacking this NLS still partially localize to nucleus, suggesting the existence of alternative determinants for FGF10′s cellular distribution [40]. Those findings are highly similar to our results, which showed the existence of a small HMW-FGF2 cytoplasmic fractionation (Fig. 1B). However, addition of NLS on 18K-FGF2 nearly forced all the overexpressed 18K-FGF2 into the nucleus, generating a more profound impact on T98G cell's proliferation compared to HMW-FGF2 (Fig. 6B).

Classical NLSs are defined as short peptide sequences that contain clusters of positively charged amino acids, typically lysine or arginine [41]. Classic NLSs are usually bound by adaptor protein such as Imp-α, which directs NLS-containing cargo complex through the nuclear pore complex (NPC) and promotes the nuclear import process [42]. Kapβ family members do not typically rely on adaptor protein binding for nuclear import, instead they directly interact with NLSs [43]. Compared to 18K-FGF2, the additional peptide sequence on HMW-FGF2 exerts function highly similar to NLS and promotes FGF2′s nuclear entry. Further examination revealed that the frequencies of glycine (G) and arginine (R) are high in this additional peptide sequence (31% for G, 17% for R), suggesting it might be a non-classical arginine-glycine-rich NLS (RG-NLS) [44]. Results presented in this study showed that Kapβ2 physically interacts with HMW-FGF2 but not 18K-FGF2 (Fig. 1C). This interaction is further proved to play a pivotal role in mediating HMW-FGF2′s nuclear localization and cellular proliferation. As Kapβ2 is also shown to interact with non-classic NLS such as RG-NLS, our data strongly support the notion that the HMW-FGF2 contains RG-NLS domain that directly interacts with Kapβ2 for nuclear import, the process of which is simultaneously facilitated by Ran GTPase.

As an important mitogenic signaling downstream of FGF2, PI3K/Akt pathway contributes to a variety of pro-survival and proliferative, as well as anti-apoptotic signaling events [45]. High expression of FGFs is correlated with activated Akt signaling in several cancer types including prostate adenocarcinoma and small-cell lung cancer, indicating that PI3K/Akt signaling cascade mediates FGF's mitogenic effects [46, 47]. We demonstrated that HMW-FGF2 overexpression is associated with reduced expression of PTEN, the negative regulator of PI3K/Akt pathway, leading to activated Akt signaling, which might be responsible for enhanced proliferative activity of T98G cells. Addition of NLS on 18K-FGF2 forced its nuclear import and activated Akt to an even higher degree than HMW-FGF2 overexpression, suggesting that nuclear import signal on FGF2 is important for the activation of its downstream mitogenic pathway. To our surprise, overexpression of 18K-FGF2, the FGF2 isoform dominantly residing in the cytoplasm, also led to reduction of PTEN level and activation of PI3K/Akt pathway. While the detailed molecular mechanism remains to be further elucidated, our findings emphasize the importance of FGF2 in the regulatory machinery governing PTEN/PI3K/Akt signaling cascade.

In conclusion, the present study investigated the cellular localization of 1ow molecular weight FGF2 isoform, 18K-FGF2 and HMW-FGF2 in T98G GBM cells. Our results demonstrated for the first time that Kapβ2 and Ran GTPase collectively facilitate the nuclear import of HMW-FGF2 via interacting with HMW-FGF2′s additional peptide sequence, which functions as a non-classic NLS. Together, this study provides novel insights to the pathogenesis and treatment of GBM.

## MATERIALS AND METHODS

### Cell culture

The human glioblastoma cell line T98G was obtained from ATCC. Cells were cultured in MEM-α medium (Invitrogen, USA) supplemented with 10% FBS and maintained at 37°C in a humidified atmosphere with 5% CO_2_. The medium used throughout this study is devoid of FGF2 and EGF.

### Antibodies

HA and Ran antibodies were obtained from Abcam (ab18181, ab157213). Kapβ2 antibody was obtained from Santa Cruz Biotechnology (sc-11368). GAPDH and histone antibody were purchased from Cell signaling Technology (2118, 8135). PTEN, p-AKT and total AKT antibodies were purchased from Millipore, USA (04-035, 05-1003, 16-294).

### Plasmid construction

FGF2 cDNA sequence (NCBI Gene ID: 2247) was used as template of PCR. Primer pair used to amplify HMW-FGF2 ORF: 5′-CCC AGA TCT ATG TAC CCA TAT GAT GTT CCA GAT TAC GCT CTG GGG GAC CGC GGG CGC GGC CGC-3′ (forward) and 5′-CCC TCT AGA TCA GCT CTT AGC AGA CAT TGG AAG-3′ (reverse). Primer pair used to amplify 18K-FGF2 ORF: 5′-CCC AGA TCT ATG TAC CCA TAT GAT GTT CCA GAT TAC GCT ATG GCA GCC GGG AGC ATC ACC ACG-3′, and same reverse primer was used. The above amplified ORF was cloned into pCMV2 vector [48] using BglII and XbaI. Peptide translation of N-terminally HA-tagged HMW-FGF2 ORF: YPYDVPDYA-LGDRGRGRALPGGRLGGRG RGRAPERVGGRGRGRGTAAPRAAPAARGSRPGPA GTMAAGSITTL PALPEDGGSGAFPPGHFKDPKRL YCKNGGFFLRIHPDGRVDGVREKSDPHIKLQLQAE ERGV VSIKGVCANRYLAMKEDGRLLASKCVTDEC FFFERLESNNYNTYRSRKYTSWYVALKRTGQYKL GSKTGPGQKAILFLPMSAKS. Peptide translation of N-terminally HA-tagged 18K-FGF2 ORF: YPYDVPDYA-MAAGSITTLPALPEDGGSGAFPPGHF KDPKRLYCKNGGFFLRIHPDGRVDGVREKSDPHIK LQLQAEERGVVSIKGVCANRYLAMKEDGRLLASK CVTDECFFFERLESNNYNTYRSRKYTSWYVALKRT GQ YKLGSKTGPGQKAILFLPMSAKS.

### Immunofluorescence

T98G cells were grown on coverslips and transfected with 18K or HMW FGF2 constructs. 24 hours after transfection, cells were washed with PBS, fixed in 4% paraformaldehyde for 45 min and then blocked and permeabilized for 90 min. Primary anti-HA antibody were incubated for 90 min followed by incubation of secondary antibody for 60 min. Stained cells were analyzed using confocal fluorescence microscopy.

### Nuclear and cytosolic fractionation

T98G cells were incubated in buffer containing 5 mM sodium phosphate, pH 7.4, 50 mM NaCl, 150 mM sucrose, 5 mM KCl, 2 mM dithiothreitol, 1 mM MgCl_2_, 0.5 mM CaCl_2_, and 0.1% digitonin. Lysates were extracted via scrapping. Nuclei fractionation was obtained by centrifuging through buffer (2.5 mM Tris-HCl, pH 7.4, 10 mM NaCl) containing 30% sucrose at 1000 g for 10 min. The supernatant was used as cytoplasmic fraction.

### Immunoprecipitation and immunoblotting

Whole cell lysates were extracted from FGF2-transfected T98G cells. Cells were washed with PBS and lysed in RIPA lysis buffer (20 mM Tris-HCl, pH 7.6, 150 mM NaCl, 1% Triton X-100 and 2 mM PMSF) with protease and phosphatase inhibitors, which were added upon use. Protein concentration was quantified by BCA assay system (Biorad, USA). 25 μg of total protein extracts were loaded for electrophoresis. Primary HA antibody was added to the whole cell protein lysates according to manufacturer's instruction and incubated at 4°C overnight. Protein beads A/G were added to pull down the HA antibody complex followed by centrifuge down the beads and boiling above 95°C. Supernatant lysates were loaded and subjected to immunoblotting analysis.

### Enzyme-linked immunosorbent assay (ELISA)

T98G cells were transfected with HMW-FGF2, 18K-FGF2 and empty vector and the culture medium were then collected for the measurement of FGF2 concentration by FGF2 ELISA kit (Abcam, USA). Detailed protocol was based on manufacturer's instructions. A series of FGF2 concentrations (0, 5, 10, 15, 20 ng/ml) were used to plot the standard curve. The FGF2 concentrations within the culture medium collected from different experimental groups were determined by applying the optical density value to the standard curve.

### RNA interference

SiRNA duplex targeting Karyopherin-β2 is synthesized by Dharmacon, GE Healthcare. T98G cells were cultured in serum-free medium and siRNA was transfected using Lipofectamine 2000 (Invitrogen, USA). Subsequent analysis was conducted 48 hours after siRNA transfection.

### Quantitative real-time PCR

RNA was isolated using Trizol reagent (Invitrogen, USA). Reverse transcription was performed using RT kit from Promega. SYBR green system (Thermo Scientific, USA) was used to conduct the real-time PCR experiment. Gene expressions were normalized to GAPDH. 2^−ΔΔCt^ method was used to analyze data.

### Treatments of GDPβS and GTPγS

T98G cells were transfected with HA-HMW-FGF2. Following transfection, culture medium was replaced by fresh medium containing 0.1 nM GTPγS or GDPβs or sterile water as control. Cells were incubated for 30 mins under 37°C, followed by whole cell lysate extraction or nuclear-cytoplasmic fractionation for western blot analysis. *MTT assay* - For the measurement of cell proliferation, 3-(4, 5-dimethyl-2-thiazoyl)-2, 5-diphenyltetrazolium bromide (MTT) was used according to manufacturer's instructions (Roche, Switzerland). T98G cells were incubated with 0.5 mg/ml MTT in the incubator with 5% O_2_ at 37°C, and then incubated with lysis buffer overnight in the incubator. The optical density of solubilized formazan was measured at 570 nm on a plate reader (BioRad, USA).

### Statistical analysis

Results were collected as the average of at least five independent experiments. All the data were presented by the mean ± SEM. The statistical significance of the difference between the values of control and treatment groups was determined by Student *t* test. Values of *p* < 0.05 were considered statistically significant.

## SUPPLEMENTARY TABLES


