# Supplementary material for: Nuclear translocation of fibroblast growth factor-2 (FGF2) is regulated by Karyopherin-β2 and Ran GTPase in human glioblastoma cells
Source: Oncotarget. 2015 May 27;6(25):21468–78. doi: 10.18632/oncotarget.4097 (PMC4673279; doi:10.18632/oncotarget.4097)
Supplement: Supplementary file 1 [file oncotarget-06-21468-s001.pdf]

SUPPLEMENTARY TABLES

Supplementary Table S1. OD readings of media with reference FGF2 concentrations from 0 to 20 ng/mL.

Reference FGF2 concentration (ng/mL)	OD reading
0	0.027 ± 0.013
0.625	0.081 ± 0.034
1.25	0.178 ± 0.048
2.5	0.383 ± 0.037
5	0.739 ± 0.044
10	1.524 ± 0.052
20	2.876 ± 0.049

Supplementary Table S2. Calculated FGF2 concentration from OD readings of three experimental cell cultures transfected with empty vector, HMW-FGF2 or 18K-FGF2 respectively. Data are shown as mean ± SEM of three independent experiments. n.s. not significant vs empty vector.

Plasmid transfected	OD reading	Calculated FGF2 concentration (ng/mL)
Empty vector	0.046 ± 0.016	0.198 ± 0.010
HMW-FGF2	0.059 ± 0.020 (n.s.)	0.288 ± 0.018
18K-FGF2	0.081 ± 0.024 (n.s.)	0.440 ± 0.045
